# Spatiotemporal control of mitoribosome-mediated metabolic reprogramming in cancer: implications for heterogeneity and therapeutic targeting

**DOI:** 10.3389/fcell.2026.1840428

**Published:** 2026-08-21

**Authors:** Xiaojie Chen, Weidong Wang, Fangxin Shen, Zhiwei Zhao

**Affiliations:** 1 The First Affiliated Hospital, College of Clinical Medicine of Henan University of Science and Technology, Luoyang, China; 2 School of Basic Medical Sciences of Henan University of Science and Technology, Luoyang, China; 3 Tuberculosis, The School of Public Health of Nanjing Medical University, The Second Hospital of Nanjing, Nanjing, China

**Keywords:** cancer metabolic reprogramming, mitoribosomes, MRPs, spatial heterogeneity, targeted therapy, temporal heterogeneity

## Abstract

Mitochondrial ribosomes (mitoribosomes), particularly mitochondrial ribosomal subunit proteins (MRPS), are emerging as contributors to cancer metabolic reprogramming. Rather than static components of mitochondrial translation, MRPS exhibit pronounced spatiotemporal heterogeneity that shapes tumor metabolic plasticity and therapeutic response. This review systematically summarizes evidence that MRPS functions are dynamically regulated across tumor progression and spatial microenvironments. Temporally, MRPS mediate metabolic switching between oxidative phosphorylation (OXPHOS) and glycolysis, contributing to metabolic adaptation, treatment resistance, and tumor evolution. Spatially, MRPS display context-dependent functions across tumor regions, cancer types, and metabolic microenvironments, thereby contributing to intratumoral metabolic diversity. We further highlight that MRPS-associated metabolic plasticity is linked with lactate metabolism and hypoxia-inducible factor (HIF) signaling, forming feedback networks associated with tumor growth, immune escape, and therapy resistance. This spatiotemporal regulatory axis challenges the traditional static view of mitochondrial dysfunction in cancer. Targeting MRPS-associated metabolic adaptation may provide therapeutic opportunities beyond the traditional Warburg framework. Emerging technologies, including lactate-sensitive nanoprobes, reactive oxygen species (ROS)-responsive delivery systems, and MRPS-related imaging platforms, may support metabolic monitoring and targeted therapeutic intervention. Collectively, this framework links mitochondrial translation with tumor metabolism and microenvironmental regulation, providing additional insight into metabolic adaptation in cancer.

## Graphical Abstract

**Figure:**
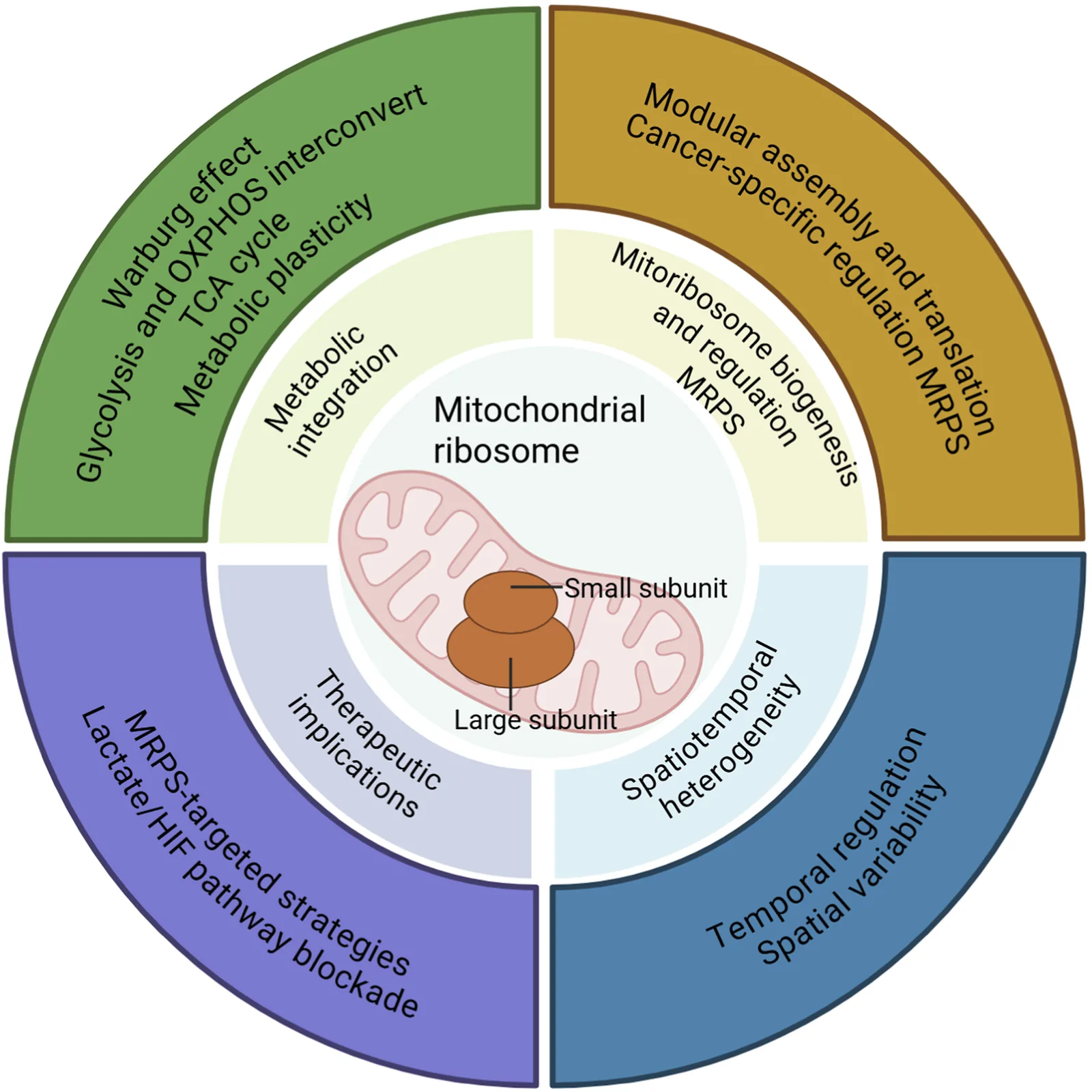
Mitochondrial ribosome-mediated cancer metabolic reprogramming This graphical abstract illustrates the role of mitoribosomes (especially MRPS) in regulating cancer metabolism. MRPS biogenesis and function are context-dependent, showing temporal and spatial heterogeneity that bidirectionally interacts with tumor metabolic states. Glycolysis and OXPHOS are not mutually exclusive; they can evolve together to support cancer cell adaptation. TCA cycle intermediates act as cofactors for epigenetic modification and as substrates for energy and macromolecule synthesis. This framework highlights metabolic plasticity and guides potential therapeutic strategies based on tumor metabolic vulnerabilities.

## 1 Introduction

Metabolic reprogramming is a defining feature of cancer that enables tumor cells to adapt to stress, sustain proliferation, and support progression (Wang Q. et al., 2025; Li L. et al., 2020; DeBerardinis et al., 2008; Tsai et al., 2023; Gong et al., 2022). Mitoribosomes are specialized mitochondrial ribonucleoprotein complexes responsible for synthesizing the 13 core subunits of oxidative phosphorylation (OXPHOS) complexes, thereby supporting mitochondrial respiratory function. Dysregulation of mitoribosomal components has been associated with altered mitochondrial translation efficiency and cancer metabolic adaptation (Wu et al., 2024; Krall et al., 2021; Du et al., 2025). Among these components, mitochondrial ribosomal subunit proteins (MRPS) have emerged as important contributors to mitochondrial translational regulation and metabolic adaptation.

The classical Warburg model proposes that cancer cells preferentially rely on glycolysis despite oxygen availability (Warburg, 1956). However, increasing evidence suggests that tumor metabolism is highly dynamic, with continuous metabolic adaptation between glycolysis and OXPHOS in response to environmental and cellular stress (Jang et al., 2025; Iborra et al., 2023; Magistretti, 2014; Sanderson et al., 2018; Lundø et al., 2020; Kohl et al., 2021). Consistent with this view, mitoribosome function is increasingly recognized as adaptable rather than static.

Despite these advances, current understanding remains fragmented. Previous studies, while identifying associations between MRPS and metabolic pathway preferences, have largely remained descriptive and have not clarified how metabolic switching between glycolysis and OXPHOS is regulated at the translational level (Kim et al., 2017; Liu et al., 2025). In particular, the temporal and spatial regulation of MRPS-associated metabolic plasticity remains poorly defined.

To address these gaps, this review proposes a spatiotemporal regulatory framework for MRPS in cancer metabolism. We hypothesize that tumor metabolic states are not fixed but evolve dynamically under the influence of genetic alterations, mitochondrial translation regulation, tumor microenvironmental cues, and metabolic feedback loops. Accordingly, we systematically examine MRPS-driven metabolic reprogramming from both temporal and spatial perspectives, focusing on mitoribosome biogenesis and translation, re-evaluation of the Warburg effect, regulation by tricarboxylic acid (TCA) cycle intermediates, and integration with major signaling pathways (Lee et al., 2014; Poznanski et al., 2021; Xi et al., 2026; Wu et al., 2021).

In this review, we discuss mitoribosomes not as static components of mitochondrial translation, but as dynamic contributors to tumor metabolic adaptation across temporal and spatial contexts. From this perspective, mitoribosome-associated metabolic plasticity may help connect mitochondrial translation, metabolic remodeling, treatment response, and tumor progression within a more integrated framework.

We focus on mitoribosome biogenesis and translation, metabolic adaptation between glycolysis and OXPHOS, integration with major signaling pathways, and the influence of tumor microenvironmental heterogeneity. We further discuss how this spatiotemporal perspective may help explain the limitations of conventional metabolic therapies and highlight emerging approaches for metabolic monitoring and therapeutic intervention (Vasan et al., 2020; Zhao et al., 2025; Xiao et al., 2023; Rios Garcia et al., 2017; Gao et al., 2025).

By linking mitochondrial translation with tumor metabolic plasticity and microenvironmental adaptation, this review provides an integrated framework for understanding MRPS function in cancer and its potential therapeutic relevance (Dupuy et al., 2015; Hulea et al., 2018; Cai et al., 2024; Pedretti et al., 2025; Chen et al., 2025; Sun et al., 2024; Brooks, 2018; Li X. et al., 2023). This integrative perspective is further developed in the section “Mitoribosomes, Metabolic Plasticity, and Tumor Adaptation”. As summarized in the Graphical Abstract, mitoribosomes, particularly MRPS, act within a context-dependent network that integrates mitochondrial translation with tumor metabolic states, supporting metabolic plasticity across glycolysis and OXPHOS and contributing to tumor adaptation.

## 2 Mitoribosome structure, biogenesis, and functional plasticity

Mitoribosomes are highly specialized mitochondrial ribonucleoprotein complexes responsible for the translation of mitochondria-encoded proteins required for OXPHOS. Their assembly, structural organization, and translational regulation are highly dynamic and closely linked to cellular metabolic states and cancer progression.

### 2.1 Structural features and modular biogenesis

**FIGURE 1:**
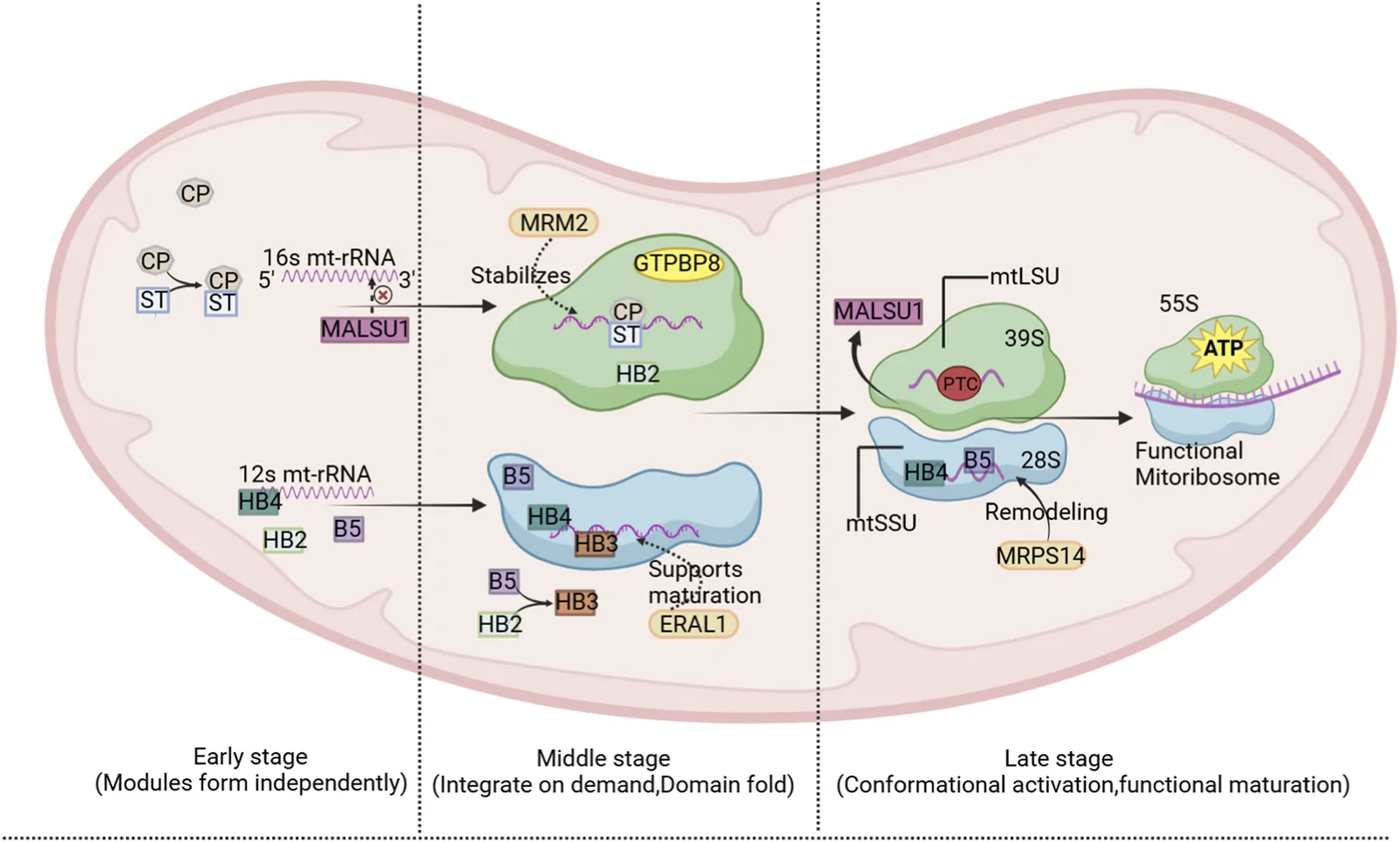
Spatiotemporal assembly of mitoribosomes. Mitoribosomes are mitochondrial ribonucleoprotein complexes responsible for translating mtDNA-encoded components of the OXPHOS system. They consist of mtLSU and mtSSU, which assemble through a modular, stepwise process involving early protein preassembly and RNA-dependent maturation. Assembly factors and quality-control proteins coordinate rRNA folding, structural stabilization, and late-stage maturation to generate a fully functional 55S mitoribosome.

Human mitoribosomes are assembled through a hierarchical and modular biogenesis process characterized by RNA-independent protein scaffolding followed by RNA-dependent maturation. During early assembly, ribosomal proteins first form discrete pre-assembled modules, including the central protuberance (CP), the stalk (ST), and body module 5 (B5) clusters. These RNA-independent protein modules exhibit relative structural stability driven by protein-protein interactions rather than rRNA scaffolding. Notably, B5 and head-body module 2 (HB2) modules can remain stable even in the absence of 12S mt-rRNA, indicating that protein-based architecture dominates early assembly steps (Figure 1) (Heinrichs et al., 2025; Park et al., 2021).

Subsequently, these pre-assembled modules sequentially dock onto the 12S mt-rRNA or 16S mt-rRNA scaffolds, initiating localized RNA folding and structural maturation. Cryo-electron microscopy studies have revealed that before module integration, key helices such as H89-H93 (helices 89–93 of mt-rRNA) remain structurally disordered. Progressive module loading induces stepwise RNA folding and drives the maturation of the ribosomal core architecture (Rebelo-Guiomar et al., 2022; Lavdovskaia et al., 2024).

RNA-binding proteins, including mitochondrial ribosomal small subunit protein14 (MRPS14), further stabilize structural transitions by promoting rRNA remodeling during module integration (Suzuki et al., 2020; Ji et al., 2024a). This modular assembly strategy compensates for reduced rRNA content in mitochondria and reflects an evolutionary shift toward protein-dominant ribosomal architecture.

### 2.2 Assembly dynamics and quality control mechanisms

Mitoribosome biogenesis is tightly regulated by multiple assembly factors and quality control checkpoints that ensure correct structural maturation and functional fidelity. Mitochondrial RNA methyltransferase 2 (MRM2) contributes to rRNA conformational maturation, particularly within the peptidyl transferase center (PTC), through structural stabilization in addition to its catalytic activity. Mitochondrial transcription termination factor 4 (MTERF4) promotes conformational ordering of H67-H71 (helices 67–71 of mt-rRNA), exposing functional domains required for assembly. Meanwhile, MRM2 stabilizes H92 (helix 92 of mt-rRNA) and promotes correct folding of H89-H90 (helices 89–90 of mt-rRNA), ensuring proper maturation of the mitochondrial large ribosomal subunit (mt-LSU).

In the absence of MRM2, mitoribosome assembly is arrested at late maturation stages, and the mitochondrial anti-associated module (MALSU1) quality control module remains bound, preventing immature ribosomal subunits from entering the translational pool. This checkpoint mechanism prevents aberrant protein synthesis but may also lead to accumulation of stalled intermediates and respiratory defects (Cipullo et al., 2021; Popay et al., 2021).

Additional factors such as GTP-binding protein 8 (GTPBP8) regulate mt-LSU maturation through interaction with 16S mt-rRNA. Loss of GTPBP8 results in accumulation of immature ribosomal intermediates and defective assembly of respiratory chain complexes, ultimately disrupting mitochondrial metabolic homeostasis (Cipullo et al., 2024). Importantly, mitoribosome assembly is not static but dynamically regulated by cellular energy status, ensuring coordination between ribosome biogenesis and metabolic demand (Schäfer et al., 2023; Singh et al., 2020).

### 2.3 Functional plasticity in translation and cancer relevance

Beyond structural assembly, mitoribosomes exhibit strong functional plasticity in regulating mitochondrial translation efficiency. Mitochondrial mRNAs lack canonical 5′ and 3′ untranslated regions, requiring specialized translation initiation mechanisms. Translation initiation is mediated by mitochondrial factors such as Pentatricopeptide Repeat Domain 3(PTCD3), which binds directly to mRNA and facilitates interaction with the small ribosomal subunit via pentatricopeptide repeat (PPR)-mediated recognition. This mechanism is distinct from cytosolic and bacterial translation systems and reflects mitochondrial-specific translational control (Rudler et al., 2019).

Mitoribosomes synthesize the core subunits of 13 OXPHOS complexes (I, III, IV, and V), and their translational efficiency is dynamically regulated according to cellular energy status (Schäfer et al., 2023; Singh et al., 2020). This enables mitoribosomes to adapt protein synthesis output in response to metabolic demands.

In cancer, mitoribosome biogenesis and translation are tightly linked to metabolic reprogramming and tumor heterogeneity (Kim and DeBerardinis, 2019). Oncogenic signaling pathways such as cellular Myelocytomatosis (c-Myc) regulate mitochondrial ribosomal proteins (e.g., mitochondrial ribosomal small subunit protein5 (MRPS5)), enhancing mitochondrial translation and promoting tumor proliferation in lung cancer and hepatocellular carcinoma. Similarly, in prostate cancer, MRPS5-mediated regulation enhances OXPHOS-dependent metabolism through c-Myc and Rat Sarcoma (Ras) signaling pathways (Markov et al., 2025; Li et al., 2021; Raimundo et al., 2012; Biffo et al., 2024). The tumor microenvironment further modulates mitoribosome function through hypoxia signaling, epigenetic regulation, and paracrine communication, dynamically controlling the balance between OXPHOS and glycolysis to support cancer cell survival under metabolic stress (Sainero-Alcolado et al., 2022; López-Otín et al., 2023; Yang et al., 2021).

Therapeutically, mitoribosomes and mitochondrial translation have emerged as potential metabolic vulnerabilities in cancer. Antibiotics targeting mitochondrial translation (e.g., tigecycline and chloramphenicol) and ETC inhibitors can disrupt OXPHOS and impair tumor growth (Vasan et al., 2020; Greenall et al., 2017; Wiederstein and Sippl, 2020; Ikeda et al., 2025; Bagaev et al., 2021; Chen et al., 2023a). However, therapeutic efficacy is often limited by metabolic compensation through glycolysis and by systemic toxicity in normal tissues dependent on mitochondrial respiration (Xiao et al., 2023).

Together, these findings suggest that mitoribosome-associated metabolic adaptation is dynamically influenced by mitochondrial stress and translational regulation. This provides the basis for the following discussion on metabolic plasticity and spatiotemporal heterogeneity in tumors.

## 3 Mitoribosome-mediated metabolic reprogramming in cancer

### 3.1 Mitoribosome dysfunction as a driver of metabolic reprogramming

Mitoribosomes are essential for the synthesis of mitochondrial DNA-encoded subunits of the respiratory chain. Deficiency of mitochondrial proteins or impaired mitoribosome assembly impairs mitochondrial translation, leading to defective ETC function and suppression of OXPHOS (Kop et al., 2019). This metabolic stress is frequently associated with compensatory upregulation of glycolysis-related pathways to maintain ATP production and survival, highlighting that mitoribosome integrity is a necessary condition for maintaining OXPHOS function (Druck et al., 2019; Nie et al., 2020; Morscher et al., 2018; Chen et al., 2022).

It should be emphasized that OXPHOS does not solely rely on pyruvate derived from glucose. Cancer cells widely utilize alternative substrates such as glutamine and fatty acids to maintain the flux of the TCA cycle. Therefore, the impairment of MRPS function leading to the compromised synthesis of respiratory chain proteins mainly affects the energy production efficiency of the electron transport chain rather than depriving the TCA cycle of its carbon source supply. The ultimate establishment of the metabolic phenotype is the result of the combined effects of mitochondrial translation capacity and multiple substrate utilization pathways.

In OXPHOS-dependent tumor subtypes, such as pancreatic cancer stem cells (Chen et al., 2026; Kessler et al., 2025; Viale et al., 2014), oxidative phosphorylation-dependent diffuse large B-cell lymphoma (OXPHOS-DLBCL) (Yang et al., 2025; Maher et al., 2026; Wu T. et al., 2025), and metastatic triple-negative breast cancer (TNBC) (Cruz-Gregorio, 2026; Malyarenko et al., 2026; Kanwal et al., 2026), mitochondrial translation factors are often upregulated to sustain respiratory activity and support tumor progression. Conversely, inhibition of mitochondrial translation disrupts respiratory chain complex assembly, blocks ATP production, and suppresses tumor growth (Martinez-Outschoorn et al., 2017; Norberg et al., 2017).

These observations indicate that metabolic imbalance between mitochondrial protein synthesis and glycolytic flux represents a key initiating event in cancer metabolic plasticity (Itoh et al., 2022; Han et al., 2024; Kamradt et al., 2021; Hu et al., 2025; Liu et al., 2017).

### 3.2 Beyond the Warburg effect: coordinating the balance between glycolysis and OXPHOS

Although the Warburg effect describes a preference for glycolysis even under oxygen availability, increasing evidence suggests that tumor metabolism is not fixed but highly dynamic (Fernández-Vizarra et al., 2022; Genuth and Barna, 2018; Reich et al., 2020; Zhu et al., 2020; Yan et al., 2023). Many cancer cells retain functional or even enhanced mitochondrial respiration, indicating that OXPHOS is not universally impaired.

Cancer cells can dynamically adjust the balance between glycolysis and OXPHOS depending on environmental and oncogenic cues, forming hybrid metabolic states rather than a strict metabolic switch. For example, inhibition of glycolysis can induce breast cancer stem cells to shift from a glycolysis-dependent quiescent state to an OXPHOS-dependent proliferative state (Wu et al., 2023; Mills et al., 2016; Morrissey et al., 2021). Similarly, SRY-Box Transcription Factor 2(SOX2)-positive prostate cancer cells exhibit simultaneous enhancement of glycolytic and mitochondrial respiratory activity, demonstrating metabolic cooperation rather than exclusivity (de Wet et al., 2022).

Notably, metabolic regulators can exert opposing functions across cancer types. Phosphoenolpyruvate Carboxykinase 1(PCK1) suppresses tumor growth in liver and kidney cancers by inducing energy crisis, but promotes colorectal and breast cancer progression through Nicotinamide Adenine Dinucleotide Phosphate (NADPH)-mediated redox adaptation. These paradoxical roles further underscore the context-dependent nature of metabolic regulation (Xiang et al., 2023; Yu et al., 2024).

**FIGURE 2:**
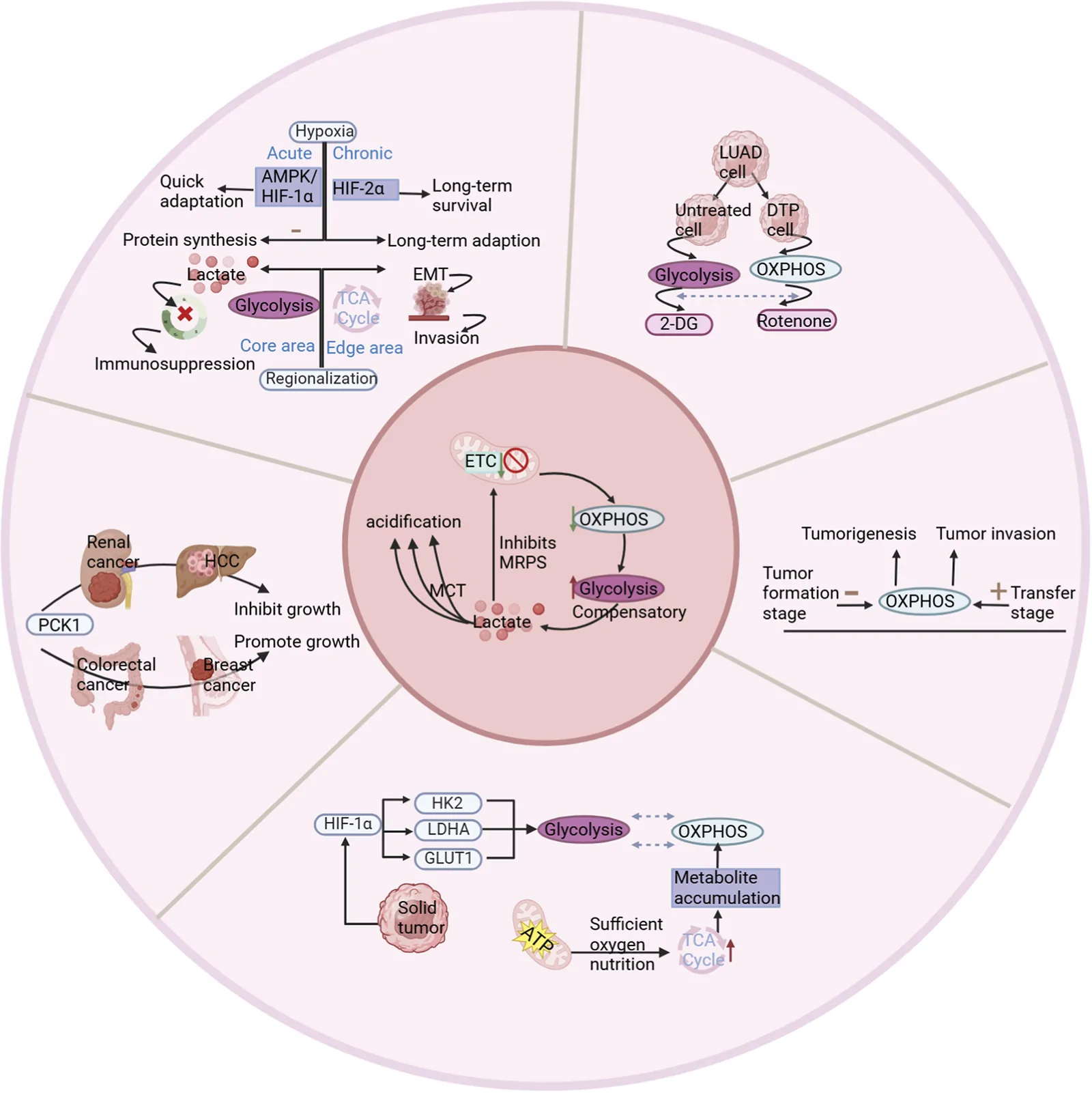
Spatiotemporal heterogeneity and metabolic feedback loops. Cancer metabolism exhibits dynamic coupling between glycolysis and OXPHOS driven by spatiotemporal heterogeneity. Impaired mitochondrial respiration promotes glycolytic reprogramming and lactate accumulation, which further suppresses mitochondrial function, forming a self-reinforcing metabolic loop. Spatially, hypoxic tumor cores favor glycolysis and immunosuppression, whereas peripheral regions retain OXPHOS activity. Temporal and cancer-type-specific metabolic switching further contributes to intratumoral heterogeneity and adaptive resistance.

These findings challenge the traditional assumption that mitochondrial dysfunction is a universal prerequisite for the Warburg phenotype and instead support a model of metabolic plasticity regulated by spatiotemporal context (Gao et al., 2019; Dong et al., 2013). Together, these findings suggest that cancer metabolism frequently exists in hybrid metabolic states, in which glycolysis and OXPHOS coexist rather than function as mutually exclusive pathways (Figure 2).

### 3.3 Metabolic intermediates as signaling mediators of mitoribosome dysfunction

Mitoribosome dysfunction–induced metabolic reprogramming extends beyond energy metabolism to involve extensive remodeling of metabolic intermediates, particularly within the TCA cycle.

Oncogenic mutations such as Kirsten Rat Sarcoma viral oncogene homolog (KRAS), MYC, Phosphoinositide 3-Kinase (PI3K) activation, or p53 loss reprogram TCA cycle substrate utilization by enhancing glycolysis and glutamine metabolism. Increased expression of glutamine transporters (e.g., Alanine-Serine-Cysteine Transporter 2) and glutaminase promotes conversion of glutamine into α-ketoglutarate (α-KG), replenishing TCA cycle intermediates and sustaining biosynthetic demand (Anderson et al., 2018; Udumula et al., 2024).

TCA cycle metabolites function as critical epigenetic regulators. α-KG serves as a cofactor for Jumonji C-domain-containing histone demethylase (JMJD) and Ten-eleven translocation methylcytosine dioxygenase (TET) DNA demethylases, promoting chromatin remodeling and gene activation (Ryan and O'Neill, 2020). In contrast, Isocitrate Dehydrogenase (IDH) mutations convert α-KG into 2-hydroxyglutarate (2-HG), which inhibits α-KG–dependent enzymes and induces epigenetic dysregulation (Martinez-Outschoorn et al., 2017). Similarly, accumulation of succinate and fumarate due to Succinate Dehydrogenase (SDH) or Fumarate Hydratase (FH) mutations inhibits α-KG–dependent dioxygenases, resulting in hypermethylation and tumorigenesis (Wang et al., 2021; Guo et al., 2024). Acetyl-CoA further promotes histone acetylation and activation of oncogenic transcriptional programs such as MYC and HIF signaling (Sletten et al., 2021; Lu et al., 2019; Yu et al., 2023).

Importantly, TCA metabolites also regulate tumor immunity. Succinate stabilizes HIF-1α and promotes M2 macrophage polarization (e.g., arginase-1, Interleukin-10), thereby suppressing anti-tumor immunity (Zhang and Xu, 2024; Li Y. et al., 2025). Tumor cells additionally compete for glucose and glutamine, inducing T cell exhaustion, while epigenetic modifications enhance Programmed Death-Ligand 1(PD-L1) expression, forming a metabolic–epigenetic–immune regulatory axis (Hong et al., 2024; Monceau et al., 2024; Chen et al., 2023b; Plebanek et al., 2024).

### 3.4 Crosstalk between mitoribosome dysfunction and HIF signaling

A key downstream consequence of mitoribosome dysfunction is activation of the HIF signaling. Impaired mitochondrial respiration disrupts ETC complexes I, IV, and V, leading to calcium imbalance and reactive ROS accumulation, which stabilizes HIF-1α signaling (Naia et al., 2023).

Stabilized HIF-1α promotes transcription of glycolytic enzymes (hexokinase 2(HK2), Phosphofructokinase-1(PFK1), Phosphoglycerate kinase 1(PGK1), Pyruvate kinase M2 isozyme (PKM2), Lactate dehydrogenase A (LDHA)) and lactate transporter Monocarboxylate transporter 4 (MCT4), while inhibiting pyruvate entry into the TCA cycle, reinforcing glycolytic flux (Zhu et al., 2024; Tommasini-Ghelfi et al., 2019). In addition, HIF suppresses Fructose-1,6-bisphosphatase 1(FBP1) and interferes with mitochondrial translation via the Mitochondrial transcription factor B1 (TFB1M)–Complex I axis, further exacerbating mitoribosome dysfunction and establishing a feed-forward loop (Wang et al., 2020; Wang H. et al., 2025; Li Z. et al., 2020).

Metabolically, accumulation of succinate and ROS inhibits prolyl hydroxylases, promoting HIF stabilization even under normoxic conditions (Barbato et al., 2023; Moss et al., 2024). This coupling positions HIF signaling as a central mediator linking mitochondrial dysfunction to metabolic reprogramming.

**FIGURE 3:**
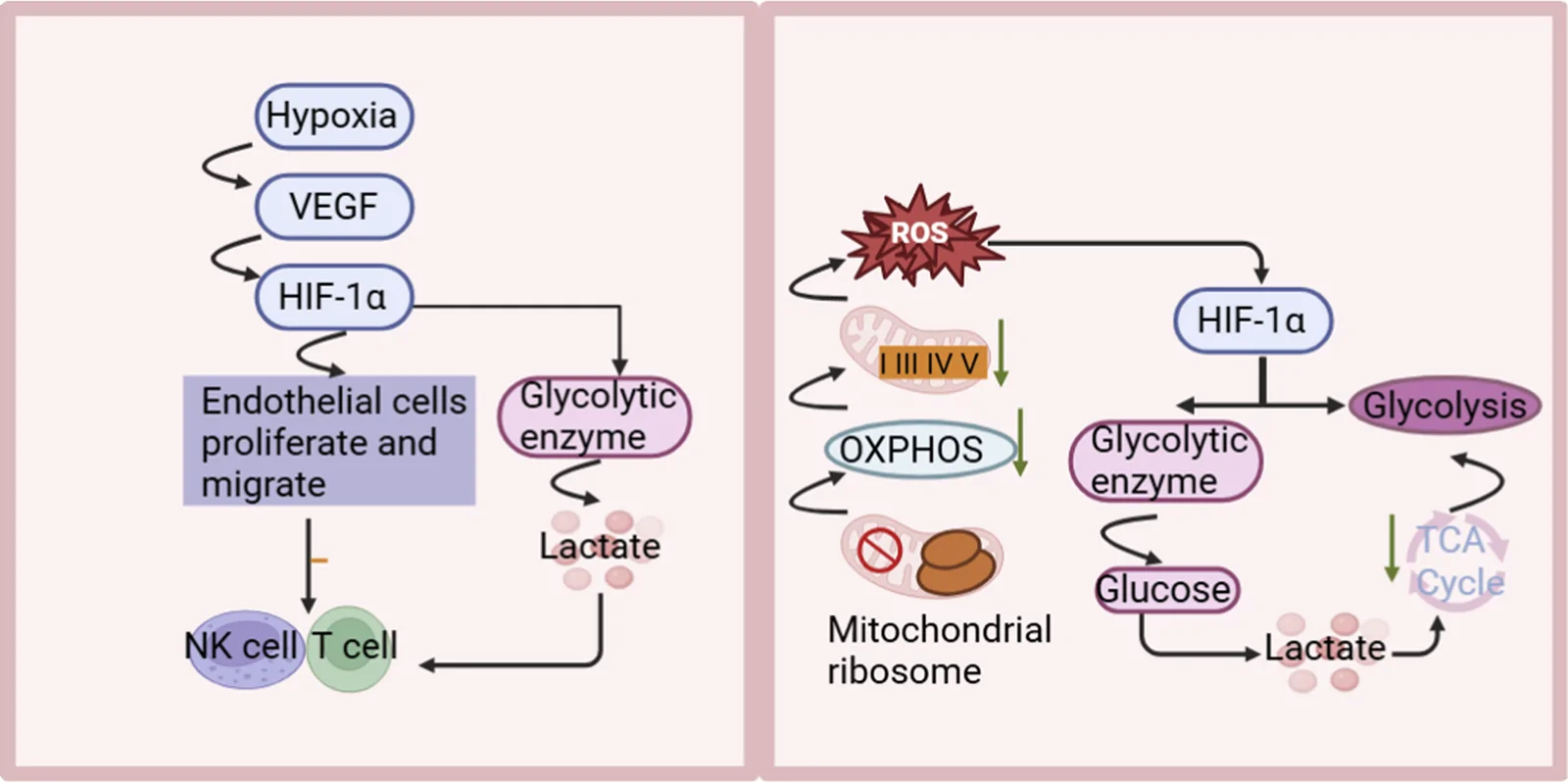
HIF-1α-mediated metabolic reprogramming and immune evasion. Mitoribosome dysfunction induces ROS-dependent stabilization of HIF-1α, promoting glycolysis, lactate accumulation, and suppression of mitochondrial metabolism. HIF-1α also drives angiogenesis and contributes to immune evasion by acidifying the tumor microenvironment and inhibiting NK and T cell activity, thereby linking metabolic reprogramming with tumor progression.

Functionally, HIF signaling also drives angiogenesis, immune escape, and tumor microenvironment remodeling, including vascular leakage, acidification, and suppression of NK and T cell function (Khalaf et al., 2021; Chen C. et al., 2023; Harris et al., 2024; Nakamura and Smyth, 2020; Sen et al., 2023; Llovet et al., 2021; Ma et al., 2025; Vecchio et al., 2025). Moreover, HIF can remain active under normoxia via lactylation-mediated stabilization, ensuring sustained metabolic adaptation (Figure 3) (Daumova et al., 2025; Elsemman et al., 2022; Luri-Rey et al., 2025).

### 3.5 Mitoribosomes as adaptive regulator of metabolic plasticity

Overall, mitoribosomes are not only the sites of protein synthesis but also the core hubs regulating the metabolic plasticity of tumors. By integrating mitochondrial translation, energy metabolism, metabolic product signaling, and hypoxia response pathways, they contribute to the regulation of metabolic adaptation by influencing the balance between OXPHOS and glycolysis, thereby shaping the adaptability of tumors to microenvironmental stress.

This mechanism highlights the status of mitochondrial ribosomes as key determinants of metabolic flexibility: by regulating the synthesis of mitochondrial-encoded proteins (such as the regulation mediated by MRPS), they directly influence the capacity and efficiency of the respiratory chain; and this regulation of OXPHOS capacity interacts with the metabolic flux directed by the Pyruvate Dehydrogenase (PDH)/Pyruvate Dehydrogenase Kinase (PDK) axis, substrate supply, and microenvironmental signals, jointly constructing the metabolic adaptability of tumors and profoundly affecting their therapeutic response. In short, mitochondrial ribosomes closely link the functional abnormalities at the organelle level with the systemic-level metabolic reprogramming and malignant progression of tumors.

Collectively, mitoribosomes act as contributors of metabolic plasticity in cancer by linking mitochondrial protein synthesis with energy metabolism and stress responses. They help control the switch between oxidative phosphorylation and glycolysis and support tumor adaptation to changes in the microenvironment. However, this regulation is not the same in all tumors or at all stages. Mitoribosome function changes with tumor location, time, and cancer type. This means a simple linear model cannot explain cancer metabolism well. Therefore, the next section discusses how mitoribosome function varies in space and time in cancer. These context-dependent metabolic adaptations provide the basis for the spatial and temporal heterogeneity of mitoribosome function discussed in the following section. Together, these observations support an integrated view of metabolic adaptation discussed in the following section.

## 4 Spatiotemporal heterogeneity of mitoribosomes in cancer

**FIGURE 4:**
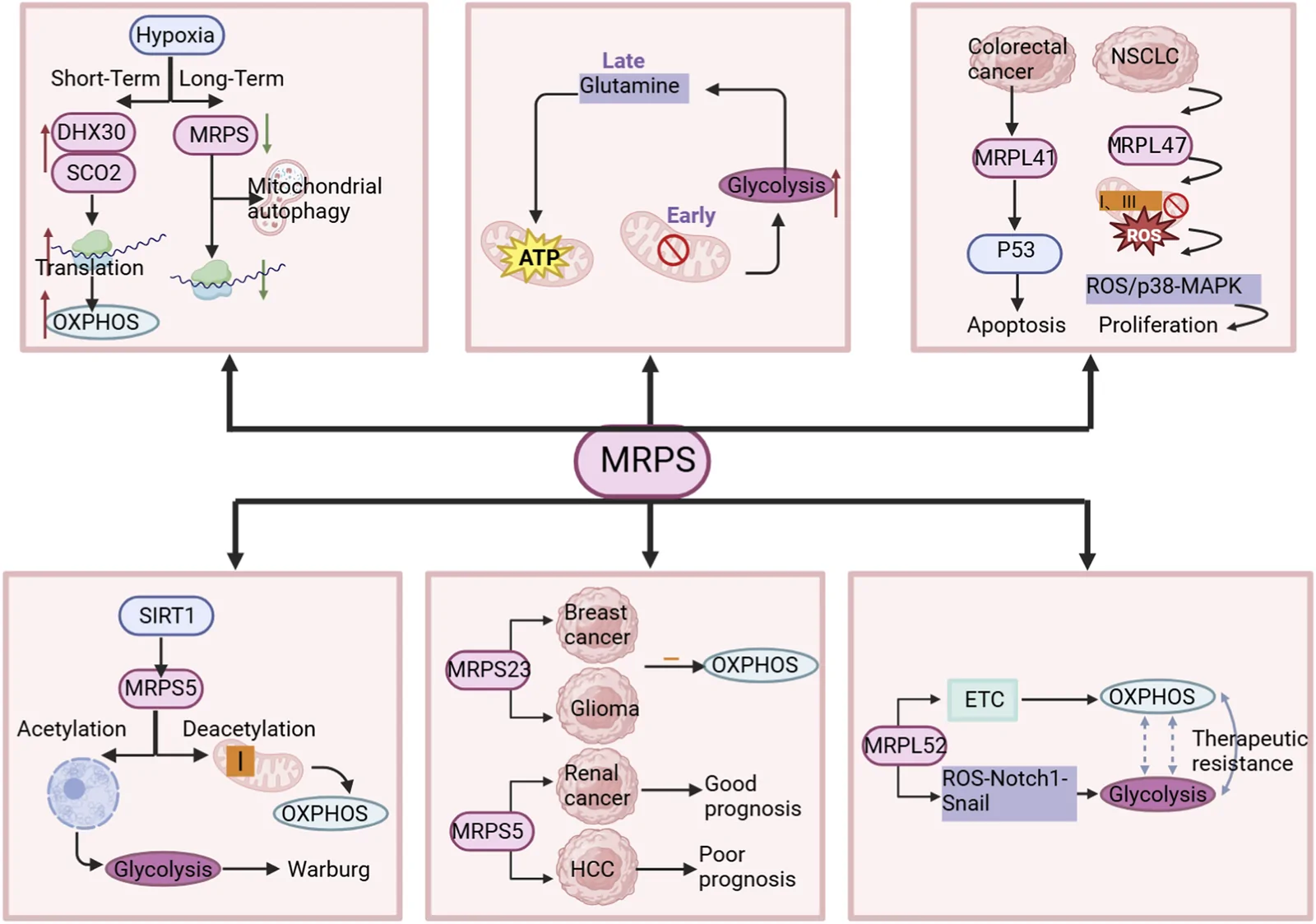
Spatiotemporal heterogeneity of mitoribosome function. Mitoribosomal proteins exhibit context-dependent regulation across time, space, and cancer types. They modulate metabolic adaptation under hypoxia, support dynamic switching between glycolysis and OXPHOS, and integrate signaling pathways such as ROS, p38-MAPK, SIRT1, and the ROS-Notch1-Snail axis. Distinct MRPS/MRPL members display opposing roles depending on tumor context, contributing to metabolic plasticity and therapeutic resistance.

Metabolic reprogramming in cancer is highly dynamic and shaped by spatial constraints, temporal evolution, and tumor-specific genetic backgrounds. Increasing evidence suggests that mitoribosome function is not static but exhibits context-dependent plasticity, contributing to metabolic heterogeneity and therapeutic resistance (Figure 4) (Sinclair et al., 2025; Woods et al., 2024; Pal et al., 2025; Yang et al., 2023; Wei et al., 2024; Peng et al., 2025; Zhang L. et al., 2024; Wu et al., 2020; Keating et al., 2020; Zhuang et al., 2023; Jiang and Ye, 2025).

### 4.1 Temporal regulation during tumor progression

Mitoribosome activity is dynamically regulated during tumor evolution in response to environmental stresses such as hypoxia and nutrient limitation. Under hypoxic conditions, tumors adjust mitochondrial translation and metabolic programs to maintain energy homeostasis, whereas prolonged stress may further shift cells toward survival-oriented metabolic states (Kim et al., 2017; Mauro et al., 2011; Yuan et al., 2023).

These temporal changes are associated with reversible transitions between glycolysis-dominant and oxidative metabolic states, reflecting metabolic flexibility rather than a unidirectional Warburg shift (Gasparre et al., 2013; Zong et al., 2016; da Silveira et al., 2020; Rosenberger et al., 2021). Overall, tumor metabolism appears to follow adaptive trajectories influenced by both genetic alterations and environmental pressures.

### 4.2 Spatial heterogeneity within tumor microenvironments

Within tumors, metabolic activity is spatially heterogeneous due to gradients of oxygen, nutrients, and stromal interactions. Hypoxic regions tend to rely more on glycolysis, whereas better-oxygenated areas retain higher oxidative metabolic activity (Lee et al., 2023; Wang et al., 2023; Donnem et al., 2025; Wang et al., 2024). This spatial compartmentalization is associated with localized regulation of mitochondrial function and metabolic adaptation.

Importantly, mitoribosomal components may contribute to these spatial differences through context-dependent regulation of mitochondrial translation and respiratory activity. Their functional effects may vary depending on cellular state and microenvironmental conditions (Wei et al., 2019; Li Y. J. et al., 2023; Liu et al., 2021; Wu H. et al., 2025).

### 4.3 Cancer-type and stage-specific functional differences

Mitoribosome-associated metabolic programs also vary across cancer types and disease stages. Tumor cells may shift between glycolysis and oxidative phosphorylation depending on tissue origin, genetic background, and treatment pressure (Kim and DeBerardinis, 2019; Li Y. et al., 2023; Derakhshan and Reis-Filho, 2022; Lee et al., 2021; Gachet et al., 2018).

During tumor progression, metabolic states are not fixed but may change dynamically in response to environmental and therapeutic stress. In some contexts, oxidative metabolism is associated with metastatic adaptation, whereas in others glycolytic programs dominate (Lee et al., 2021; Gachet et al., 2018). These cancer-type- and stage-specific metabolic preferences underscore the heterogeneous nature of tumor metabolism.

### 4.4 Integrated model of mitoribosomal spatiotemporal plasticity

Collectively, current evidence suggests that mitoribosome heterogeneity is influenced by temporal metabolic changes, microenvironmental conditions, and tumor-specific molecular features. Rather than functioning in a fixed manner, mitoribosomal proteins may exhibit different roles across metabolic states and tumor contexts.

This spatiotemporal perspective links mitochondrial translation with metabolic plasticity and tumor microenvironmental heterogeneity, providing a more integrated framework for understanding tumor metabolic adaptation in cancer.

## 5 Mitoribosomes, metabolic plasticity, and tumor adaptation

Mitoribosome-driven metabolic reprogramming not only reshapes energy metabolism but also contributes to tumor adaptation to environmental stress, immune pressure, and therapeutic intervention. By integrating mitochondrial translation, metabolic flux, and microenvironmental signals, mitoribosomes may act as important regulators linking metabolic plasticity with tumor adaptation.

### 5.1 Metabolic switching and adaptive energy reprogramming

Cancer cells exhibit highly flexible metabolic states, shifting between glycolysis-dominant and OXPHOS-supported states in response to genetic background, microenvironmental stress, and therapeutic pressure.

Defects in mitoribosome assembly impair mitochondrial respiratory chain protein synthesis and inhibit OXPHOS, forcing tumor cells to rely on glycolysis for ATP production (Kop et al., 2019; Druck et al., 2019; Nie et al., 2020; Morscher et al., 2018; Chen et al., 2022). Conversely, in OXPHOS-dependent tumors such as pancreatic cancer stem cells, diffuse large B-cell lymphoma, and triple-negative breast cancer metastases, mitochondrial translation factors are upregulated to sustain respiratory function and tumor growth (Martinez-Outschoorn et al., 2017; Norberg et al., 2017).

Metabolic reprogramming is highly reversible. Tumor cells dynamically switch metabolic states through feedback regulation between glycolysis and OXPHOS. Lactate accumulation is associated with PDK-mediated PDH inhibition, which limits pyruvate oxidation and favors glycolytic adaptation. Additionally, the transport efficiency of mitochondrial pyruvate carrier (MPC) is also involved in this process, determining the flux of pyruvate from the cytoplasm into the mitochondria, jointly constituting an important switch for the metabolic adaptability of tumors (Kuo et al., 2010; Vinay et al., 2015; Papadopoli et al., 2025; Pan et al., 2025; Zhang H. et al., 2024; Li C. et al., 2025; Zhang et al., 2025; Yang et al., 2026).

Furthermore, the metabolic symbiosis phenomenon in the tumor microenvironment is particularly crucial. In the “reverse Warburg effect” model in a specific area, cancer-associated fibroblasts (CAF) exhibit active glycolysis, generating a large amount of metabolites such as lactic acid and pyruvate. These substances are taken up by the adjacent tumor cells, which then reconvert them into pyruvate within the mitochondria, serving as an alternative carbon source to support OXPHOS.

Meanwhile, the ROS produced by tumor cells will in turn promote the glycolytic level of stromal cells, thereby establishing a bidirectional metabolic coupling system between the two (Qin et al., 2020).

Therapy-induced metabolic switching is also prominent. After KRAS inhibition in pancreatic cancer, tumor cells enhance mitochondrial biogenesis through PPARγ Coactivator-1 alpha (PGC1α) activation, while B-Raf proto-oncogene (BRAF) inhibitor-treated melanoma selects for slow-cycling, OXPHOS-dependent subpopulations with elevated mitochondrial metabolism (Yoshida, 2015; Yang et al., 2022; Lin et al., 2024; Stoldt et al., 2025).

### 5.2 Immune escape driven by metabolic reprogramming

Metabolic heterogeneity within the tumor microenvironment plays a central role in immune evasion. In hypoxic tumor regions, HIF-1α signaling strongly activates glycolysis-related genes such as Glucose Transporter 1 (GLUT1) and LDHA while suppressing mitochondrial respiration (Wallace, 2012; Li J. et al., 2025; Yang et al., 2020). This contributes to lactate accumulation, extracellular acidification, and suppression of cytotoxic T-cell activity, thereby promoting immune escape (Xia et al., 2021; Pouysségur et al., 2022).

In contrast, oxygen-rich tumor regions maintain partial OXPHOS activity supported by TCA cycle intermediates such as citrate and α-KG (Wallace, 2012; Li J. et al., 2025; Yang et al., 2020). This spatial metabolic gradient creates functional compartmentalization within tumors, where immunosuppressive cells preferentially accumulate in hypoxic cores, while effector immune cells are restricted by acidic environments. In melanoma and other solid tumors, such lactate-rich hypoxic niches are tightly associated with immune suppression, including Treg cell stabilization and inhibition of effector CD8^+^ T cell function, thereby reinforcing tumor immune escape mechanisms (Watson et al., 2021; Xiong et al., 2022; Wu Y. et al., 2025).

### 5.3 Therapy resistance and metabolic plasticity

Metabolic plasticity mediated by mitoribosomes is a major driver of therapeutic resistance. Under chemotherapy or targeted therapy, cancer cells often shift from glycolysis toward mitochondrial OXPHOS to maintain survival under metabolic stress. For example, KRAS-mutant pancreatic cancer cells increase mitochondrial biogenesis through PGC1α activation after pathway inhibition, while BRAF inhibitor-treated melanoma selects for OXPHOS-dependent, slow-cycling JARID1B (Jumonji, AT-Rich Interactive Domain 1B)-high cells (Yoshida, 2015; Yang et al., 2022; Lin et al., 2024; Stoldt et al., 2025).

More broadly, therapy-induced stress promotes activation of autophagy and lysosomal degradation pathways, supporting mitochondrial adaptation and metabolic resilience. These processes are closely linked to mitochondrial translation regulation and mitoribosomal function, supporting tumor persistence under treatment pressure (Lee et al., 2021; Gachet et al., 2018; Ji et al., 2024b; Gu et al., 2021).

### 5.4 Integrated model of tumor metabolic adaptation

Collectively, mitoribosome-driven metabolic plasticity integrates energy metabolism, microenvironmental cues, and therapeutic stress responses to promote tumor adaptation. Tumor metabolism is therefore not static but continuously reshaped by feedback loops between glycolysis, OXPHOS, and environmental stress factors such as hypoxia, lactate accumulation, and stromal interaction. Together, these adaptive interactions highlight how mitoribosome-associated metabolic plasticity contributes to tumor survival, immune escape, and therapeutic resistance.

## 6 Therapeutic implications and future perspectives

As discussed in the integrative framework above, the dynamic nature of tumor metabolic adaptation may contribute to therapeutic resistance and metabolic heterogeneity. Mitoribosome-associated metabolic adaptation links mitochondrial translation with hypoxia signaling and metabolic feedback regulation, suggesting potential points of therapeutic vulnerability. However, the complexity and spatial heterogeneity of tumor metabolism remain major challenges for effective metabolic intervention.

### 6.1 Targeting mitoribosome-mediated metabolism

Mitoribosomes contribute to mitochondrial respiratory function through regulation of mitochondrial translation. Dysregulation of mitoribosomal proteins may impair OXPHOS and promote compensatory metabolic adaptation toward glycolysis (Kop et al., 2019; Druck et al., 2019; Nie et al., 2020; Morscher et al., 2018; Chen et al., 2022; Chang et al., 2022). These changes are associated with altered mitochondrial stress responses, hypoxia signaling, and metabolic remodeling in cancer cells (Naia et al., 2023; Zhu et al., 2024; Tommasini-Ghelfi et al., 2019; Wang et al., 2020).

Current therapeutic approaches mainly focus on modulation of mitochondrial translation, mitochondrial biogenesis, and hypoxia-associated signaling pathways (Schulte et al., 2023; Mao et al., 2025). However, the metabolic heterogeneity of tumors may limit the efficacy of single-pathway interventions.

### 6.2 Lactate and metabolic intervention strategies

Lactate is closely associated with glycolytic metabolism, microenvironmental acidification, and metabolic adaptation in tumors (Sica et al., 2019; Liu W. W. et al., 2024; Liu Y. et al., 2024). Accumulation of lactate and proton transport contributes to an acidic tumor microenvironment, which may further influence mitochondrial function and metabolic flexibility (Guo et al., 2022; Xue et al., 2022; Wang and Ma, 2025; Cohen et al., 2024; Tan et al., 2021).

Metabolic interactions between stromal cells and tumor cells may further support metabolic adaptation across different tumor regions (Lin et al., 2024). Current therapeutic strategies targeting lactate metabolism mainly include inhibition of LDH activity, blockade of monocarboxylate transporters, and disruption of tumor–stromal metabolic coupling (Kuo et al., 2010; Vinay et al., 2015; Papadopoli et al., 2025; Pan et al., 2025; Zhang H. et al., 2024; Li C. et al., 2025; Zhang et al., 2025; Yang et al., 2026; Qin et al., 2020; Wallace, 2012; Li J. et al., 2025; Yang et al., 2020; Xia et al., 2021; Pouysségur et al., 2022; Watson et al., 2021; Xiong et al., 2022; Wu Y. et al., 2025).

### 6.3 Nanoplatform-based precision delivery

Because tumor metabolism exhibits strong spatial heterogeneity, metabolically responsive delivery systems have attracted increasing attention. A dual-compartment chitosan nanoparticle is engineered to exploit—and partition payloads across—the tumor periphery-core pathophysiological divide: the margin is mildly acidic (pH 6.5–6.8), perfused, and CD8^+^T cell-bearing, whereas the core is deeply hypoxic/anoxic, glycolytically addicted, and functionally isolated from uniform drug diffusion; a single-compartment, homogeneous release would therefore misdirect each agent. The peripheral arm uses chitosan’s pH-responsive protonation (pKa ∼6.3): deacetylated–NH_2_ stay deprotonated (compact) at pH 7.4 but protonate at the margin, driving matrix swelling and margin-gated release of encapsulated anti-PD-1—the chitosan shell being chosen to shield IgG from degradation/proteolysis, stabilize it in circulation, and localize checkpoint blockade where T cells actually engage the tumor. The core arm carries nitroimidazole-functionalized crosslinks that act as bioreductive gatekeepers: under deep hypoxia (≤1% O_2_), nitroreductase-mediated electron transfer irreversibly reduces the nitro group → hydroxylamine/amine conversion, cleaving crosslinks/disassembling the carrier to dump the glycolysis/LDH-targeting agent (e.g., oxamate) strictly inside the anoxic compartment.

**FIGURE 5:**
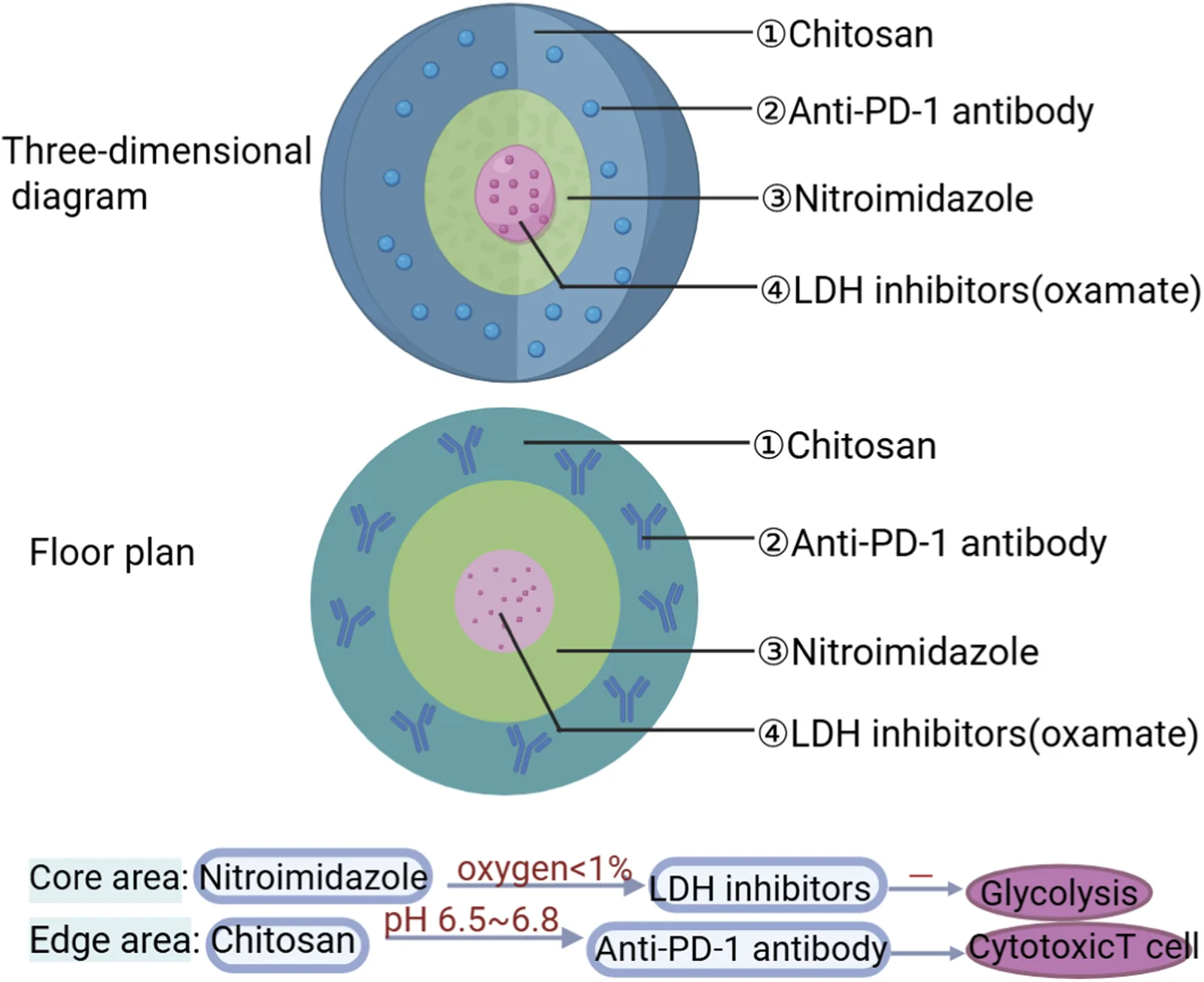
Nanoplatform-based spatially targeted therapy. A dual-compartment nanodelivery system enables spatially selective drug release based on tumor microenvironmental cues. Hypoxic tumor cores trigger hypoxia-responsive carriers to release glycolysis-targeting agents, while acidic peripheral regions activate pH-sensitive systems to release immune checkpoint inhibitors. This design enables coordinated targeting of metabolic and immune compartments while minimizing systemic toxicity. Anti-PD-1 antibody: immune checkpoint inhibitor targeting the PD-1 signaling pathway in cancer immunotherapy.

Thus, the system couples pH-triggered immune activation at the invasive front with hypoxia-triggered metabolic collapse in the core through two orthogonal, microenvironment-defined release gates (Figure 5) (Tsai et al., 2023; Xiao et al., 2023; Zou and Green, 2023; Mottas et al., 2019). Approaches based on hypoxia-, ROS-, or lactate-responsive systems may help improve regional drug delivery and metabolic intervention within tumors.

In addition, imaging-assisted metabolic monitoring may support evaluation of metabolic states during treatment. However, most of these approaches remain exploratory and require further experimental and clinical validation (Kuang et al., 2024; Liu L. et al., 2024).

### 6.4 Challenges and future directions

Despite substantial progress, several challenges remain in targeting mitoribosome-associated metabolic reprogramming. First, tumor metabolism is highly dynamic and heterogeneous. Cancer cells can switch between glycolysis and OXPHOS under therapeutic pressure, reducing the long-term efficacy of single-pathway interventions (Liu et al., 2021; Sighel et al., 2021; Stuhldreier et al., 2022; Blomberg et al., 2023).

Another challenge is the incomplete understanding of context-dependent mitoribosome function. MRPS and MRPL proteins may exhibit different roles across tumor types, disease stages, and microenvironmental conditions. Therefore, mitoribosomes should not be viewed as direct determinants of tumor metabolism, but rather as contributors to mitochondrial adaptation and respiratory regulation.

Future studies will likely require more integrated approaches combining metabolic intervention, microenvironmental regulation, and improved metabolic monitoring. Advances in spatial transcriptomics, single-cell analysis, and metabolic imaging may further improve understanding of mitoribosome-associated heterogeneity in cancer (Kuang et al., 2024; Liu L. et al., 2024).

Collectively, these efforts may help provide a more integrated understanding of mitoribosome-associated metabolic adaptation and its potential therapeutic relevance in cancer.

## Glossary

MRPS: mitochondrial ribosomal subunit proteins
OXPHOS: oxidative phosphorylation
HIF: hypoxia-inducible factor
ROS: reactive oxygen species
TCA: tricarboxylic acid
CP: central protrusion
ST: also known as the L7/L12 stalk, whose core is a heterodimer composed of mitochondrial ribosomal proteins mL48 and mL49
B5: module 5 of the body
HB2: head-body module 2
H89-H93: helices 89–93 of mt-rRNA
MRPS14: mitochondrial ribosomal small subunit protein14
MRM2: mitochondrial RNA methyltransferase 2
PTC: peptidyl transferase center
MTERF4: mitochondrial transcription termination factor 4
H67-H71: helices 67–71 of mt-rRNA
H92: helix 92 of mt-rRNA
H89–H90: helices 89–90 of mt-rRNA
Mt-LSU: mitochondrial large ribosomal subunit
MALSU1: mitochondrial anti-associated module
GTPBP8: GTP-binding protein 8
PTCD3: Pentatricopeptide Repeat Domain 3
PPR: pentatricopeptide repeat
c-Myc: cellular Myelocytomatosis
MRPS5: mitochondrial ribosomal small subunit protein5, also structurally known as uS5
Ras: rat sarcoma
ETC: electron transport chain
CRISPR-Cas13a: Clustered Regularly Interspaced Short Palindromic Repeats and CRISPR-associated protein 13a
OXPHOS-DLBCL: oxidative phosphorylation-dependent diffuse large B-cell lymphoma
TNBC: triple-negative breast cancer
SOX2: SRY-Box Transcription Factor 2
KRAS: kirsten rat sarcoma viral oncogene homolog
PI3K: Phosphoinositide 3-Kinase
α-KG: α-ketoglutarate
JMJD: Jumonji C-domain-containing histone demethylase
TET: ten-eleven translocation methylcytosine dioxygenase
IDH: isocitrate dehydrogenase
2-HG: 2-hydroxyglutarate
SDH: succinate dehydrogenase
FH: fumarate hydratase
HK2: hexokinase 2
PFK1: Phosphofructokinase-1
PGK1: Phosphoglycerate kinase 1
PKM2: pyruvate kinase M2 isozyme
LDHA: lactate dehydrogenase A
MCT4: monocarboxylate transporter 4
FBP1: fructose-1,6-bisphosphatase 1
TFB1M: mitochondrial transcription factor B1
PDH: pyruvate dehydrogenase
PDK: Pyruvate Dehydrogenase Kinase
DHX30: DEAH-box helicase 30
SCO2: synthesis of cytochrome c oxidase 2
MRPL47: mitochondrial ribosomal large subunit protein 47
NSCLC: non-small cell lung cancer
p38-MAPK: p38 Mitogen-Activated Protein Kinase
MRPL41: mitochondrial ribosomal large subunit protein 41
EMT: epithelial-mesenchymal transition
SIRT1: sirtuin 1, NAD-dependent deacetylase
MRPS23: mitochondrial ribosomal small subunit protein 23
MRPL52: mitochondrial ribosomal large subunit protein 52
ROS-Notch1-Snail: Reactive Oxygen Species-Notch signaling receptor 1-Snail family transcriptional. ROS upregulates Notch1, increasing Snail transcription to promote EMT and metastasis
GLUL: glutamate-ammonia ligase
2-DG: 2-deoxy-d-glucose
PCK1: phosphoenolpyruvate carboxykinase 1
NADPH: nicotinamide adenine dinucleotide phosphate
MPC: mitochondrial pyruvate carrier
CAF: cancer-associated fibroblasts
PGC1α: PPARγ Coactivator-1 alpha
BRAF: B-Raf proto-oncogene
GLUT1: glucose transporter 1
JARID1B: Jumonji, AT-Rich Interactive Domain 1B
LDH: lactate dehydrogenase
MCT1: monocarboxylate transporter 1
anti-PD-1: anti-programmed cell death protein 1 antibody
MRPS/MRPL: mitochondrial ribosomal small subunit protein/mitochondrial ribosomal large subunit protein
CRISPR: clustered regularly interspaced short palindromic repeats
mt-LSU: mitochondrial large ribosomal subunit
mt-SSU: mitochondrial small ribosomal subunit
VEGF: vascular endothelial growth factor
